# Expressional analysis of the astrocytic Kir4.1 channel in a pilocarpine–induced temporal lobe epilepsy model

**DOI:** 10.3389/fncel.2013.00104

**Published:** 2013-07-05

**Authors:** Yuki Nagao, Yuya Harada, Takahiro Mukai, Saki Shimizu, Aoi Okuda, Megumi Fujimoto, Asuka Ono, Yoshihisa Sakagami, Yukihiro Ohno

**Affiliations:** Laboratory of Pharmacology, Osaka University of Pharmaceutical SciencesOsaka, Japan

**Keywords:** Kir4.1 channel, astrocytes, temporal lobe epilepsy, status epilepticus, spatial potassium buffering, pilocarpine

## Abstract

The inwardly rectifying potassium (Kir) channel Kir4.1 in brain astrocytes mediates spatial K^+^ buffering and regulates neural activities. Recent studies have shown that loss-of-function mutations in the human gene *KCNJ10* encoding Kir4.1 cause epileptic seizures, suggesting a close relationship between the Kir4.1 channel function and epileptogenesis. Here, we performed expressional analysis of Kir4.1 in a pilocarpine-induced rat model of temporal lobe epilepsy (TLE) to explore the role of Kir4.1 channels in modifying TLE epileptogenesis. Treatment of rats with pilocarpine (350 mg/kg, i.p.) induced acute status epilepticus, which subsequently caused spontaneous seizures 7–8 weeks after the pilocarpine treatment. Western blot analysis revealed that TLE rats (interictal condition) showed significantly higher levels of Kir4.1 than the control animals in the cerebral cortex, striatum, and hypothalamus. However, the expression of other Kir subunits, Kir5.1 and Kir2.1, remained unaltered. Immunohistochemical analysis illustrated that Kir4.1-immunoreactivity-positive astrocytes in the pilocarpine-induced TLE model were markedly increased in most of the brain regions examined, concomitant with an increase in the number of glial fibrillary acidic protein (GFAP)-positive astrocytes. In addition, Kir4.1 expression ratios relative to the number of astrocytes (Kir4.1-positive cells/GFAP-positive cells) were region-specifically elevated in the amygdala (i.e., medial and cortical amygdaloid nuclei) and sensory cortex. The present study demonstrated for the first time that the expression of astrocytic Kir4.1 channels was elevated in a pilocarpine-induced TLE model, especially in the amygdala, suggesting that astrocytic Kir4.1 channels play a role in modifying TLE epileptogenesis, possibly by acting as an inhibitory compensatory mechanism.

## INTRODUCTION

The spatial K^+^ buffering by astrocytes removes excess extracellular K^+^ at synapses and transports them into regions of low K^+^ concentration such as blood vessels, regulating neuronal activities (Walz, 2000; Kofuji and Newman, 2004; Simard and Nedergaard, 2004; Butt and Kalsi, 2006). The K^+^ buffering currents are mediated by inwardly rectifying potassium (Kir) channels which are expressed in astrocytes (Tanemoto et al., 2000; Hibino et al., 2004; Kofuji and Newman, 2004; Simard and Nedergaard, 2004; Butt and Kalsi, 2006). These comprise Kir4.1 channels, homo-tetramers of Kir4.1 subunits, and Kir4.1/5.1 channels, hetero-tetramers of Kir4.1 and Kir5.1 subunits, which conduct large inward K^+^ currents at potentials negative to K^+^ equilibrium potential (Tanemoto et al., 2000; Ohno et al., 2007; Su et al., 2007; Furutani et al., 2009). In addition, spatial K^+^ buffering is linked to glutamate uptake and/or aquaporin-4-mediated water transport by astrocytes (Nagelhus et al., 1999; Amiry-Moghaddam and Ottersen, 2003; Puwarawuttipanit et al., 2006; Djukic et al., 2007; Kucheryavykh et al., 2007).

Recent clinical studies have shown that mutations in the human gene *KCNJ10* encoding Kir4.1 cause EAST (epilepsy, ataxia, sensorineural deafness, and tubulopathy) or SeSAME (seizures, sensorineural deafness, ataxia, mental retardation, and electrolyte imbalance) syndrome consisting of generalized tonic-clonic (GTC) seizures, ataxia, hearing loss, and abnormal renal excretion of electrolytes (Bockenhauer et al., 2009; Scholl et al., 2009). The most frequent mutation of *KCNJ10 *was R65P at the cytoplasmic end of transmembrane region (TM)-1 and others include G77R (TM-1), C140R (extracellular loop between TM-1 and TM-2), T164I, A167V (cytoplasmic end of TM-2), R175Q, R199X, and R297C (C-terminal domain; Reichold et al., 2010; Sala-Rabanal et al., 2010; Tang et al., 2010). All these mutations caused drastic decreases in K^+^ currents mediated by Kir4.1 and Kir4.1/5.1 channels, suggesting that the impaired functioning of astrocytic Kir4.1 channels causes epileptic seizures by disrupting spatial K^+^ buffering. In addition, several SNPs of *KCNJ10* have been shown to be associated with temporal lobe epilepsy (TLE) with febrile seizures (Heuser et al., 2010). Expressional analysis also revealed pathophysiological alterations in Kir4.1 expression in patients with TLE (Das et al., 2012; Heuser et al., 2012; Steinhäuser et al., 2012), suggesting a potential involvement of Kir4.1 channels in TLE epileptogenesis. However, information on the modulatory role of Kir4.1 in the generation and/or development of TLE is still very limited.

In the present study, we performed expressional analysis of Kir4.1 in a pilocarpine-induced rat model of TLE to explore the pathophysiological role of Kir4.1 channels in TLE epileptogenesis. The expressions of Kir5.1 and Kir2.1, other Kir subunits expressed in astrocytes, were also evaluated for comparison.

## RESULTS

### PILOCARPINE-INDUCED TLE MODEL

All the TLE rats (N = 11) used herein experienced pilocarpine (350 mg/kg, i.p.)-induced status epilepticus (repeated and sustained clonic seizures) and showed spontaneous seizures (i.e., wild running/jumping and GTC seizures) 7–8 weeks after the pilocarpine treatment. The animals, which were given pilocarpine but did not experience status epileptics and any seizure activity thereafter (7–8 weeks), were used as the control (*N *= 11). Four and seven animals in each group were subjected to Western blot and immunohistochemical analysis, respectively.

### WESTERN BLOT ANALYSIS

**FIGURE 1:**
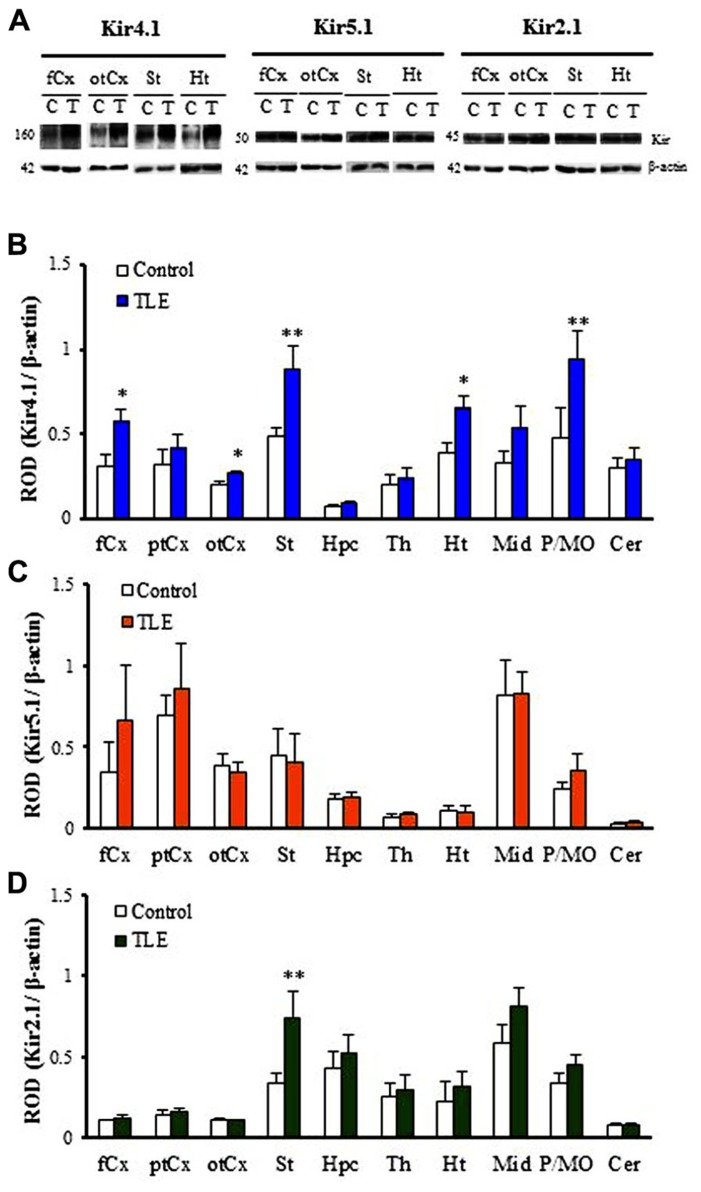
**Western blot analysis for Kir4.1, Kir5.1 and Kir2.1 expression in pilocarpine-inducedTLE rats.** **(A)** Representative Western blots visualizing Kir4.1, Kir5.1, and Kir2.1 expression in the frontal cortex (fCx), occipito-temporal cortex (otCx), striatum (St), and hypothalamus (Ht). **(B–D)** Regional expression of Kir4.1 **(B)**, Kir5.1 **(C)**, and Kir2.1 **(D)** in pilocarpine-induced TLE rats. Kir expression was expressed as relative optical density (ROD) to β-actin. fCx, frontal cortex; ptCx, parieto-temporal cortex; otCx, occipito-temporal cortex; St, striatum; Hpc, hippocampus; Th, thalamus; Ht, hypothalamus; Mid, midbrain; P/MO, pons/medulla oblongata; Cer, cerebellum. Each column represents the mean ± SEM of four animals. *P <0.05, **P <0.01, significantly different from the control rats.

As reported previously (Connors et al., 2004; Seifert et al., 2009; Harada et al., 2013), Kir4.1 was detected primarily as a tetramer (~160 kDa) in all brain regions examined in TLE and control rats (**Figure 1A**). Two-way ANOVA revealed no significant interaction [*F*(1, 60) = 1.61, *P* = 0.13], but significant main effects of groups [*F*(1, 60) = 23.24, *P* < 0.01] and regions [*F*(9, 60) = 10.80, *P* < 0.01]. Expression levels of Kir4.1 were relatively high in the striatum (St) and pons/medulla oblongata (P/MO). As compared to control animals, TLE rats showed significantly higher Kir4.1 levels in the frontal cortex (fCx, *P* < 0.05), occipito-temporal cortex (otCx,*P* < 0.05), St (*P* < 0.01), hypothalamus (Ht,*P* < 0.05), and P/MO (*P* < 0.01; **Figures 1A, B**). These changes were region-specific and the Kir4.1 levels in other brain regions [i.e., parieto-temporal cortex (ptCx), hippocampus (Hpc), thalamus (Th), midbrain (Mid), and cerebellum (Cer)] remained unaltered.

In contrast to Kir4.1, Kir5.1 and Kir2.1 subunits were detected mainly as monomers (Kir5.1: 50 kDa, Kir2.1: 45 kDa) in all 10 regions (**Figure 1A**). Levels of Kir5.1 were relatively high in the ptCx and Mid while the Kir2.1 levels were high in the Mid and low in the cerebral cortices and Cer (**Figures 1C, D**). Analysis of Kir5.1 expression showed only a significant main effect of regions [*F*(9, 60) = 7.97, *P* < 0.01] without a significant interaction [*F*(9, 60) = 0.32, *P* = 0.96] or a main effect of groups [*F*(9, 60) = 0.77, *P* = 0.38]. Thus, no significant differences in the expression levels of Kir5.1 were observed between TLE and control rats in all 10 regions (**Figure 1C**). On the other hand, analysis of Kir2.1 expression revealed significant main effects of groups [*F*(1, 60) = 7.93, *P* < 0.01] and regions [*F*(9, 60) = 13.9, *P* < 0.01] without a significant interaction [*F*(9, 60) = 1.24, *P* = 0.29]. Among 10 regions, only the Kir2.1 level in the St was significantly (*P* < 0.01) higher in TLE than in control rats (**Figure 1D**).

### IMMUNOHISTOCHEMICAL ANALYSIS FOR KIR4.1 EXPRESSION

**FIGURE 2:**
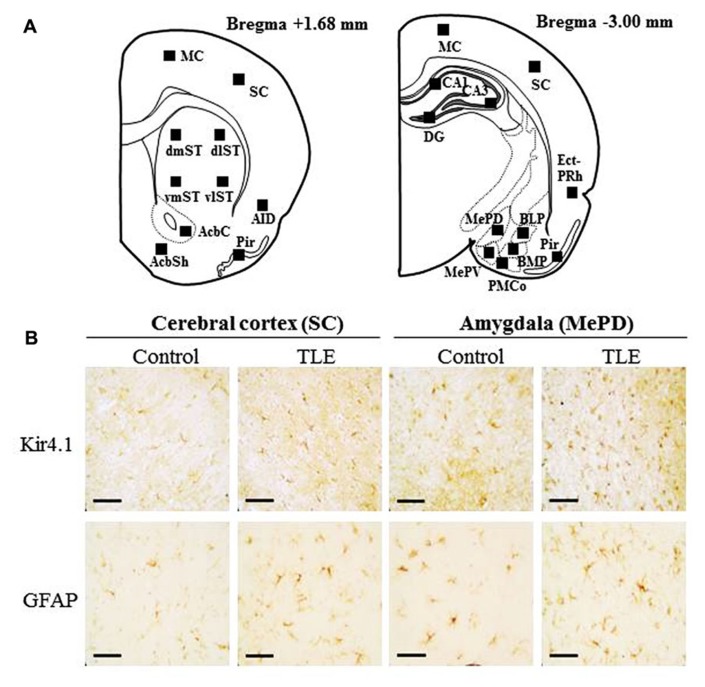
**Immunohistochemical analysis of Kir4.1- and GFAP-immunoreactivity (IR)-positive cells in pilocarpine-inducedTLE rats.** **(A)** Schematic illustrations of the brain sections selected for quantitative analysis of Kir4.1- and GFAP-IR-positive cells. Squares in each section indicate the area analyzed for counting of Kir4.1- and GFAP-IR-positive cells. The distance from the Bregma is shown on the bottom of each section. MC, motor cortex; SC, sensory cortex; AID, agranular insular cortex; Pir, piriform cortex; dmST, vmST, dlST and vlST, dorsomedial, ventromedial, dorsolateral, and ventrolateral striatum, respectively; AcbC and AcbSh, core and shell regions of nucleus accumbens, respectively; Ect-PRh, ectorhinal–perirhinal cortex; MePV and MePD, medial amygdaloid nucleus, posteroventral and posterodorsal part; BLP, basolateral amygdaloid nucleus, posterior part; BMP, basomedial amygdaloid nucleus, posterior part; PMCo, posteromedial cortical amygdaloid nucleus; CA1, CA3, and DG: CA1, CA3, and dentate gyrus of the hippocampus. **(B)** Representative photographs illustrating the Kir4.1 (upper panels)- and GFAP (lower panels)-positive cells in the sensory cortex (SC) and the medial amygdaloid nucleus, posterodorsal part (MePD). Scale bar: 50 μ m.

**Figure A1:**
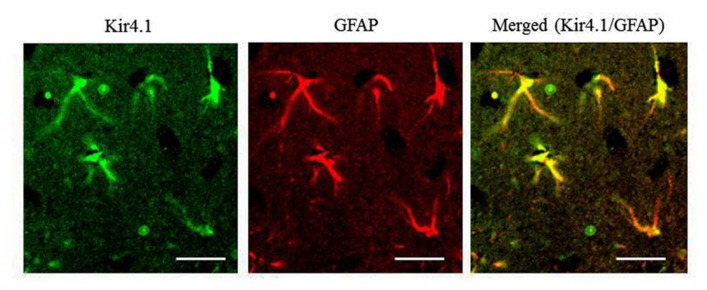
**Expressional patterns of Kir4.1 in the rat hippocampus.** Typical photograph illustrating a double staining of Kir4.1 with GFAP in the hippocampal CA1 field. The hippocampal section was incubated anti-GFAP antibody (Progen, Heidelberg, Germany) for 24 h at 4°C and then incubated with a FITC (fluorescein isothiocyanate; green fluorescence)-conjugated goat anti-rabbit IgG secondary antibody (Sigma-Aldrich) or TRITC (tetramethylrhodamine-5- (and 6)-isothiocyanate; red fluorescence)-conjugated goat anti-mouse IgG secondary antibody (Sigma-Aldrich) to probe Kir4.1 and GFAP, respectively. Immunofluorescence images were obtained with a confocal laser scanning microscope. Scale bar: 50 μm. All the Kir4.1-immunoreactivity (IR)-positive cells with the stellate-shape were double-stained with anti-GFAP antibody (yellow in a merged picture). Kir4.1-IR was occasionally found in a few round-shaped cells with no GFAP-IR [also negative to a neuronal marker, neuronal nuclear antigen (NeuN)], which were omitted from the analysis.

Since Western blot analysis revealed that pilocarpine-induced TLE rats showed elevated Kir4.1 expression in the fCx and otCx, we further conducted immunohistochemical analysis for Kir4.1 expression using frontal (Bregma +1.68 mm level) and occipito-temporal (Bregma -3.00 mm level) brain slices (**Figure 2A**). With regard to the expression patterns of Kir4.1-immunoreactivity (IR), we have previously shown that Kir4.1 was primarily stained in astrocytes which typically show a stellate-shape and were specifically co-stained with glial fibrillary acidic protein (GFAP; an astrocyte marker; Harada et al., 2013; also see **Figure A1** in Appendix). Although Kir4.1-IR was also found in a small population of round-shaped (small) cells, which might possibly represent oligodendrocyte precursor cells (Maldonado et al., 2013), we omitted them from the analysis and solely counted the stellate-shaped astrocytes probe with anti-Kir4.1 antibody. In addition, to evaluate changes in the total number of astrocytes *per se* and the Kir4.1 expression ratio relative to the total number of astrocytes, we also performed immunohistochemical analysis of GFAP using paired successive slices obtained from the same animal.

**FIGURE 3:**
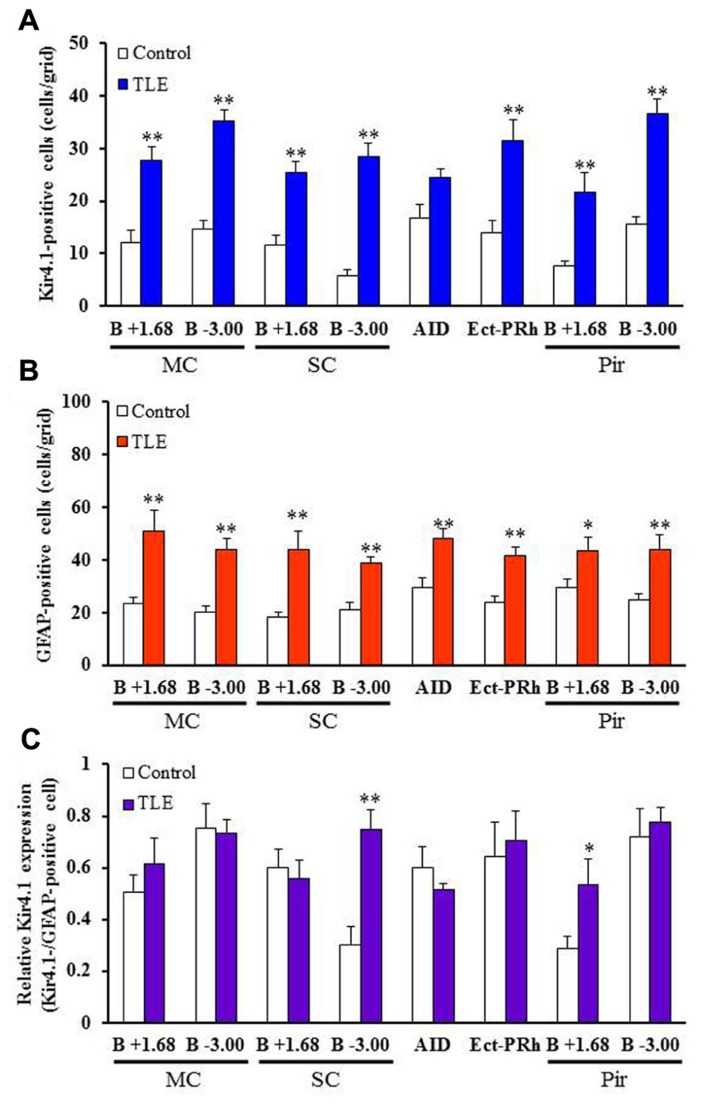
**Topographical expression of Kir4.1 and GFAP in the cortical regions of pilocarpine-inducedTLE rats.** **(A,B)** Number of Kir4.1 **(A)**- or****GFAP **(B)**-immunoreactivity (IR)-positive cells. **(C)** Relative Kir4.1 expression ratios in astrocytes. A pair of successive slices in each region from the same animal was stained with anti-Kir4.1 or anti-GFAP antibody. The Kir4.1 expression ratios were calculated as the ratios of Kir4.1-positive astrocytes relative to the total number of astrocytes (Kir4.1-positive cells/GFAP-positive cells) in each animal. MC, motor cortex; SC, sensory cortex; AID, agranular insular cortex, dorsal part; Ect-PRh, ectorhinal-perirhinal cortex; Pir, piriform cortex. Each column represents the mean ± S.E.M. of seven animals. *P <0.05, **P < 0.01, significantly different from control rats.

**FIGURE 4:**
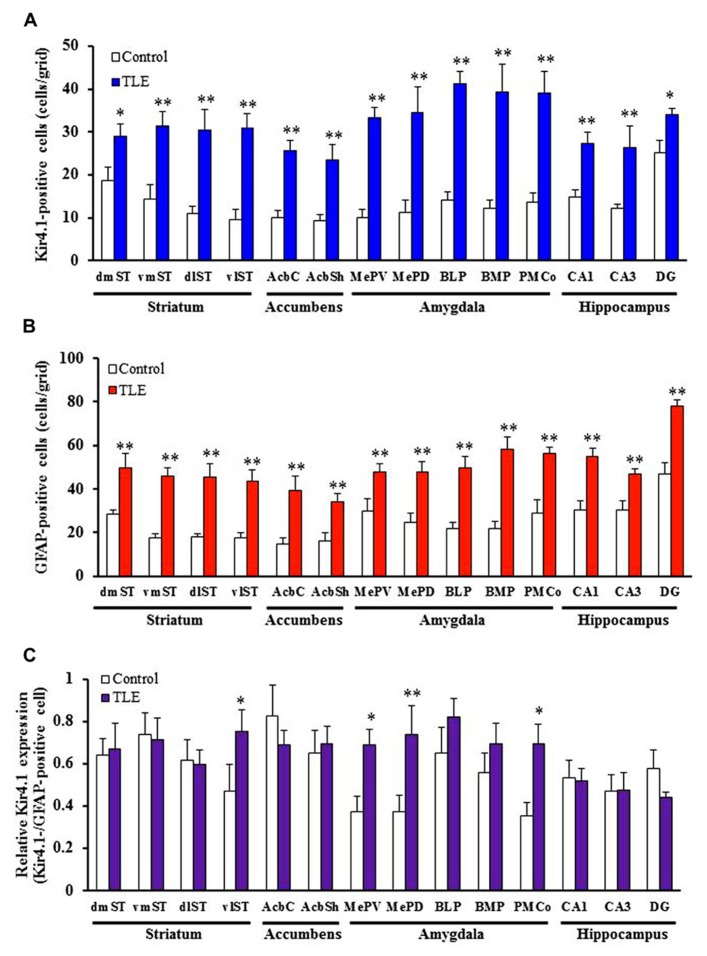
**Topographical expression of Kir4.1 and GFAP in the basal ganglia and limbic regions of pilocarpine-inducedTLE rats.** **(A,B)** Number of Kir4.1 **(A)**- or GFAP **(B)**-immunoreactivity (IR)-positive cells. **(C)** Relative Kir4.1 expression ratios in astrocytes. A pair of successive slices in each region from the same animal was stained with anti-Kir4.1 or anti-GFAP antibody. The Kir4.1 expression ratios were calculated as the ratios of Kir4.1-positive astrocytes relative to the total number of astrocytes (Kir4.1Kir4.1-positive cells/GFAP-positive cells) in each animal. dmST, vmST, dlST, and vlST, dorsomedial, ventromedial, dorsolateral, and ventrolateral striatum, respectively; AcbC and AcbSh, core and shell regions of the nucleus accumbens, respectively; MePV and MePD, medial amygdaloid nucleus, posteroventral and posterodorsal part; BLP, basolateral amygdaloid nucleus, posterior part; BMP, basomedial amygdaloid nucleus, posterior part; PMCo, posteromedial cortical amygdaloid nucleus; CA1, CA3, and DG, CA1, CA3, and dentate gyrus of the hippocampus. Each column represents the mean ± SEM of seven animals. *P < 0.05, **P 0.01, significantly different from control rats.

In accordance with previous studies (Connors et al., 2004; Seifert et al., 2009; Harada et al., 2013), Kir4.1 was mostly expressed in stellate-shaped cells (**Figure 2B**). Two-way ANOVA revealed significant interaction groups × regions [*F*(21, 264) = 1.91, *P* < 0.05] and significant main effects of groups [*F*(1, 264) = 410.45, *P* < 0.01] and regions [*F*(21, 264) = 3.50, *P* < 0.01]. In pilocarpine-induced TLE rats, Kir4.1 expression was significantly elevated in all brain regions examined [dentate gyrus of the Hpc (DG) and dorsomedial St (dmST): *P* < 0.05, other regions: *P* < 0.01] except for the agranular insular cortex dorsal part (AID; **Figures 2– 4**). The number of Kir4.1-IR-positive astrocytes increased two to four times the control levels in TLE animals and these changes were prominent in the sensory cortex (SC), lateral St, and amygdala (**Figures 3A and 4A**). In addition, the number of GFAP-IR-positive astrocytes *per se* also increased in pilocarpine-induced TLE rats (**Figures 3B and 4B**). Analysis of GFAP expression showed significant main effects of groups [*F*(1, 264) = 333.16, *P* < 0.01] and regions [*F*(21, 264) = 6.26, *P* < 0.01] without a significant interaction [*F*(1, 264) = 0.86, *P* = 0.65]. The numbers of GFAP-IR-positive cells in all 22 brain regions examined were significantly [piriform cortex (Pir) B+1.68: *P* < 0.05, other regions: *P* < 0.01] higher in TLE than in control rats. We then compared the Kir4.1 expression ratios relative to the number of astrocytes (Kir4.1-IR-positive cells/GFAP-IR-positive cells). Two-way ANOVA revealed significant interaction [*F*(21, 264) = 1.78, *P* < 0.05] and significant main effects of groups [*F*(1, 264) = 12.82, *P* < 0.01] and regions [*F*(21, 264) = 2.36, *P* < 0.01]. The relative Kir4.1 expression ratios in astrocytes were 0.3–0.8 in most regions of the brain in the control animals (0.557 ± 0.022), but the values were significantly (*P* < 0.01) increased in TLE group (0.652 ± 0.019). These changes were region-specific and significant increases were observed in the posteroventral (MePV, *P* < 0.05) and posterodorsal (MePD, *P* < 0.01) parts of the medial amygdaloid nucleus, the posteromedial cortical amygdaloid nucleus (PMCo, *P* < 0.05), ventrolateal St (vlST, *P* < 0.05), SC (*P* <0.01), and Pir (*P* < 0.05; **Figures 3C and 4C**).

## DISCUSSION

Temporal lobe epilepsy is the most common type of partial complex seizure in adulthood (Hauser et al., 1996; Wieser, 2004). The main features of TLE include (1) localization of seizure foci in the limbic structures (e.g., Hpc and amygdala), (2) existence of a “latent period,” a seizure-free time interval following the initial precipitating injury, (3) incidence of mesial sclerosis leading to atrophy (e.g., neuronal loss and gliosis) in the limbic structures (Mathern et al., 1997; Bartolomei et al., 2005; Curia et al., 2008). The pilocarpine-induced TLE model shares important features of human TLE such as (1) presence of a latent period followed by spontaneous recurrent seizures, (2) occurrence of wide spread brain injuries resembling human TLE, (3) similarity of drug responses to human TLE (e.g., relatively resistant to conventional antiepileptics; Leite et al., 1990; Cavalheiro et al., 1991; Glien et al., 2002; Löscher, 2002; Wieser, 2004; Chakir et al., 2006; Curia et al., 2008). The present study demonstrated for the first time that expression of astrocytic Kir4.1 channels mediating spatial K^+^ buffering was markedly elevated in a pilocarpine-induced TLE model. The elevation of Kir4.1 expression in the TLE model was characterized by the following points, (1) subunit-specificity for Kir4.1, (2) a partial association with an increase in the number of astrocytes (i.e., astrogliosis) and (3) the most prominent elevation in the amygdala.

In this study, Western blot analysis revealed that the pilocarpine-induced TLE model exhibits a subunit-specific increase in the Kir4.1 expression with negligibly affecting the level of Kir5.1 and Kir2.1 subunits. Kir5.1 subunits, like Kir4.1, are expressed in astrocytes and form heteromeric Kir4.1/5.1 channels with Kir4.1, mediating K^+^ buffering (Tanemoto et al., 2000; Hibino et al., 2004; Kofuji and Newman, 2004). In contrast, Kir2.1 subunits are predominantly expressed in neurons to regulate the resting membrane potential while several reports show that astrocytes also express Kir2.1 to some degree in several brain regions (e.g., Pir and olfactory bulb; Howe et al., 2008; Kang et al., 2008). Our results suggest that, among astrocytic Kir channels, Kir4.1 channels play the most important role in modulating TLE epileptogenesis.

Elevation of Kir4.1 expression in the pilocarpine-induced TLE model was widely spread throughout brain regions examined and these changes were generally associated with an increase in the number of astrocytes, which was probably due to astrogliosis following status epilepticus-induced brain injury (Leite et al., 1990; Cavalheiro et al., 1991; Borges et al., 2003; Curia et al., 2008). Although astrogliosis may also contribute to epileptogenesis, it can compensate abnormal discharges and promote tissue repair. Astrocytes can reduce abnormal neural excitation by spatial buffering of potassium and by taking up synaptically released glutamate. In addition, they can secrete growth factors [e.g., glial cell line-derived neurotrophic factor (GDNF) and nerve growth factor (NGF)] and cytokines (e.g., TNF-α) that mediate neuronal survival, axonal/dendritic sprouting, and homeostatic plasticity (Borges et al., 2003; Fellin, 2009). Thus, the up-regulation of Kir4.1 associated with status epilepticus-induced astrogliosis might negatively regulate the TLE epileptogenesis by normalizing extracellular K^+^ ([K^+^]_o_) and glutamate ([glutamate]_o_). Furthermore, significantly higher Kir4.1 expression ratios relative to the number of astrocytes (Kir4.1-IR-positive cells/GFAP-IR-positive cells) were observed region-specifically in the amygdaloid nuclei (i.e., MePV, MePD, and PMCo). These results illustrate the important role of amygdalar Kir4.1 channels in modifying status epileptics-induced epileptogenicity in TLE. Since deficit or knockdown of astrocytic Kir4.1 channels is known to impair K^+^- and glutamate-uptake into astrocytes and facilitate seizure generation (Djukic et al., 2007; Kucheryavykh et al., 2007; Bockenhauer et al., 2009; Scholl et al., 2009; Reichold et al., 2010; Sala-Rabanal et al., 2010; Tang et al., 2010), up-regulation of Kir4.1 channels in the pilocarpine TLE model seemed to occur as a compensatory mechanism to the limbic hyperexcitability in TLE epileptogenesis. Indeed, the medial amygdaloid and cortical amygdaloid nuclei are known to be closely linked to kindling epileptogenesis and human epileptic disorders including TLE (Hosford et al., 1995; Morimoto et al., 2004). Although it is known that pilocarpine-induced status epilepticus causes neural damage, sclerosis, and rewiring not only in the amygdala, but also in the Hpc, changes in the relative Kir4.1 expression ratios were not significant in the Hpc (i.e., CA2). This may be due to the relatively low expression level of Kir4.1 in the Hpc as compared to other brain regions (see **Figure 2**).

Evidence is accumulating that dysfunction of astrocytic Kir4.1 channels is causative of seizure activity generation. Specifically, loss-of-function mutations in human Kir4.1 gene (*KCNJ10*) cause the EAST syndrome, including GTC seizures and ataxia (Bockenhauer et al., 2009; Scholl et al., 2009; Reichold et al., 2010; Sala-Rabanal et al., 2010; Tang et al., 2010). It is also suggested that the down-regulation of Kir4.1 expression in the amygdala is related to seizure induction in an animal model of GTC seizures (Harada et al., 2013). Furthermore, recent studies showed the down-regulation and/or impaired functioning of Kir4.1 channels in specimens from patients with TLE (Das et al., 2012; Heuser et al., 2012; Steinhäuser et al., 2012), suggesting a close relationship of Kir4.1 to human TLE. The present results (Kir4.1 up-regulation) in the pilocarpine-induced TLE model, however, were different from the findings of Kir4.1 expression (Kir4.1 down-regulation) in patients with TLE. Although the reasons for this discrepancy are currently uncertain, it may result from the difference in the etiological basis between human TLE and pharmacologically evoked seizure. In fact, we also observed that Kir4.1 expression in the paralimbic cortex was gradually increased during the kindling development induced by pentylentetrazole (Mukai et al., 2013). Alternatively, it may be due to the temporal changes in Kir4.1 expression. Since the present study analyzed the Kir4.1 expression shortly after the occurrence of spontaneous seizures, the down-regulation of Kir4.1 may occur at a more advanced (delayed) stage in the pilocarpine-induced TLE model. Indeed, a recent study showed that Kir4.1 expression was down-regulated by local inflammatory events after TLE-associated brain injury, implying that the down-regulation of Kir4.1 could be a consequence, and not a primary cause, of seizures (Zurolo et al., 2012). Further studies are required to delineate the time course of the Kir4.1 expressional changes and the mechanisms underlying the Kir4.1 up-regulation in the pilocarpine-induced TLE model.

In conclusion, we performed expressional analysis of Kir4.1 in a pilocarpine-induced rat model of TLE to explore the pathophysiological role of Kir4.1 channels in epileptogenesis. Western blot analysis revealed that Kir4.1 levels of TLE rats under an interictal state were significantly increased in the cerebral cortex, St, and Ht while the levels of other Kir subunits, Kir5.1 and Kir2.1, were unaltered. Immunohistochemical analysis demonstrated that TLE rats showed a widespread elevation in Kir4.1 expression which accompanied an increase in the number of astrocytes *per se*. In addition, the Kir4.1 expression ratio relative to the increase in the astrocyte number was also elevated region-specifically in the amygdaloid nuclei in a pilocarpine TLE model. The present findings suggest that astrocytic Kir4.1 channels play a modulatory role in TLE epileptogenesis, possibly by acting as an inhibitory compensatory mechanism. Further studies using patch-clamp and/or microdialysis techniques are necessary to delineate the functional alterations (e.g., changes in Kir4.1-mediated potassium currents, extracellular levels of K^+^ and glutamate) of up-regulated Kir4.1 channels in the TLE model.

## MATERIALS AND METHODS

### ANIMALS

Male SD rats (7 weeks old; Japan SLC, Shizuoka, Japan) were used. Animals were kept in air-conditioned rooms under a 12-h light/dark cycle (light on: 6:00 AM) and allowed *ad libitum* access to food and water. The housing conditions of the rat and animal care methods complied with the NIH guide for the care and use of laboratory animals. The experimental protocols of this study were approved by the Experimental Animal Research Committee at Osaka University of Pharmaceutical Sciences.

### PILOCARPINE-INDUCED TLE MODEL

A pilocarpine-induced TLE model was prepared according to methods reported previously (Cavalheiro, 1995; Liu et al., 2008). Briefly, animals were first treated with methyl-scopolamine (1 mg/kg, i.p., Sigma-Aldrich, St. Louis, MO, USA) to reduce peripheral cholinergic side effects and, 30 min later, pilocarpine (350 mg/kg, i.p., Sigma-Aldrich) was injected to induce acute status epilepticus. Pilocarpine-induced status epilepticus was then terminated by the injection of diazepam (10 mg/kg, i.p., CERCINE^®^ INJECTION, Takeda Pharmaceutical Co. Ltd., Osaka, Japan) at 5, 20, 80, 300, and 420 min after the onset of status epilepticus (repeated and sustained clonic seizures). Animals which did not show any seizure activity (status epileptics) within 20 min after the pilocarpine injection were used as the control and treated with diazepam in the same manner as the status epilepticus-experienced rats. All animals were fed for 7–8 weeks after the pilocarpine treatment. Eleven out of the twelve rats which experienced pilocarpine-induced status epilepticus showed spontaneous seizures, (i.e., wild running/jumping and GTC seizures) and were defined as TLE rats. One animal which showed pilocarpine-induced status epilepticus but did not any spontaneous seizure was excluded from the analysis. None of the control animals (*N * = 11) showed any seizures or abnormal behavior during the 7–8 weeks observation period.

### WESTERN BLOT ANALYSIS

Temporal lobe epilepsy rats under interictal conditions (*N * = 4) or control rats (*N * = 4) were deeply anesthetized with pentobarbital (80 mg/kg, i.p.). The brain was then removed from the skull, chilled in ice-cold saline and dissected into the following 10 regions (fCx, ptCx, otCx, St, Hpc, Th, Ht, Mid, P/MO, and Cer). Brain samples were then homogenized in an ice-cold lysis buffer (pH 7.5) containing: (in mM) Tris 20, NaCl 150, MgCl_2_ 10, EDTA 1.0, EGTA 1.0, 1% Triton X-100, and a mixture of protease inhibitors (leupeptin, aprotinin, E-64, pepstatin A, bestatin, and 4-(2-aminoethyl) benzenesulfonyl fluoride hydrochloride; Nacalai Tesque, Kyoto, Japan). The homogenate was centrifuged at 15,000*g*, 4°C for 30 min and the supernatant was stored at -80°C for the Western blot analysis.

Western blots were performed as published previously (Ohno et al., 2009; Harada et al., 2013). Briefly, samples were incubated with a sodium dodecyl sulfate-polyacrylamide gel electrophoresis (SDS-PAGE) sample buffer for 5 min at 95°C. Each sample (40 μg/lane) was then subjected to SDS-PAGE and separated proteins were transferred for 60 min to a PVDF membrane (GE Healthcare, Buckinghamshire, UK). The membrane was first incubated with a blocking solution containing 0.3–2% skim milk, 25 mM Tris, 150 mM NaCl, and 0.1% Tween 20 (pH 7.5) for 60 min, then with the corresponding primary antibodies overnight (4°C), followed by a 60 min-incubation with the secondary antibody, a goat anti-rabbit IgG-HRP conjugate (1:2000, Santa Cruz Biotechnology, CA, USA) for Kir4.1, a donkey anti-goat IgG-HRP conjugate (1:2000, Santa Cruz Biotechnology) for Kir5.1 or Kir2.1, or a sheep anti-mouse IgG-HRP conjugate (1:2000, GE Healthcare) for β-actin. The primary antibodies used were a rabbit polyclonal antibody against Kir4.1 (1:500, Alomone Labs., Jerusalem, Israel), a goat polyclonal antibody against Kir5.1 (N-12; 1:400, Santa Cruz Biotechnology), a goat polyclonal antibody against Kir2.1 (1:400, Santa Cruz Biotechnology) and mouse monoclonal antibodies against β-actin (1:1000, Sigma-Aldrich). Final detection was performed with the enhanced chemiluminescence methodology (Amersham ECL Western blotting detection reagents and analysis system, GE Healthcare) using a lumino imaging analyzer (LAS-3000, FUJIFILM, Tokyo, Japan). To normalize for protein loading, chemiluminescence of the bands in each lane was standardized to the intensity of the β-actin band in the same lane.****

### IMMUNOHISTOCHEMICAL ANALYSIS

Brains were obtained from TLE rats (interictal status; *N * = 7) or control rats (*N * = 7) in the same manner as for the Western blot analysis. After fixation in a 4% paraformaldehyde solution for 24 h, brain samples were dehydrated and embedded in paraffin. Formalin-fixed and paraffin-embedded tissue samples were cut into 4-μm thick sections and a pair of successive slices in each brain region was immunohistochemically stained with anti-Kir4.1 or anti-GFAP antibody using the avidin–biotin complex (ABC) method (Ohno et al., 2009,^2012^; Harada et al., 2013). Briefly, the fronto- and occipito-temporal brain sections were deparaffinized in xylene and then rehydrated in ethanol. Sections were autoclaved for 10 min to retrieve the antigen. After cooling to room temperature, endogenous peroxidase activity was quenched by 3% H_2_O_2_ and non-specific binding was blocked using a 5% skim milk solution. Sections were then incubated overnight (4°C) with a rabbit anti-Kir4.1 antibody (1:100, Alomone Labs) and a mouse anti-GFAP antibody (1:100, Progen) in the 5% skim milk solution. Thereafter, they were incubated with a biotinylated goat anti-rabbit IgG secondary antibody (1:400, Vector Laboratories, Burlingame, CA, USA) and a goat anti-mouse IgG secondary antibody (1:400, Sigma-Aldrich) for 60 min and with an avidin-biotinylated horseradish peroxidase complex (Vectastain ABC Kit) for an additional 60 min. Kir4.1- and GFAP-IR was visualized by the diaminobenzidine–nickel staining method.

The number of Kir4.1- or GFAP-IR-positive cells was counted in a 350 × 350 μm^2^ grid laid over various regions of the brain (**Figure 4**), which included the following regions: the motor cortex (MC), SC, AID, ectorhinal–perirhinal cortex (Ect-PRh), Pir, dorsolateral St (dlST) and dmST, vlST and ventromedial St (vmST), core (AcbC) and shell (AcbSh) regions of the nucleus accumbens, MePV, MePD, basolateral amygdaloid nucleus posterior part (BLP), basomedial amygdaloid nucleus posterior part (BMP), PMCo, and CA1, CA3, and the DG of the Hpc. Relative expression rate of Kir4.1 was defined as a percentage of the number of Kir4.1-positive cells relative to that of GFAP-positive cells.

### STATISTICAL ANALYSIS

All data are expressed as the mean ± SEM. Expressional changes in Kir channel subunits determined by Western blot or immunohistochemical analysis were compared by two-way ANOVA followed by Tukey multiple comparison test. Differences were considered to be statistically significant for values of *P* < 0.05.

## Conflict of Interest Statement

The authors declare that the research was conducted in the absence of any commercial or financial relationships that could be construed as a potential conflict of interest.

## ABBREVIATIONS

GFAP: glial fibrillary acidic protein
GTC: generalized tonic-clonic
Kir: inwardly-rectifying potassium
SE: status epilepticus
TLE: temporal lobe epilepsy
